# Supplementary material for: A new twist in the photophysics of the GFP chromophore: a volume-conserving molecular torsion couple
Source: Chem Sci. 2018 Jan 10;9(7):1803–12. doi: 10.1039/c7sc04091a (PMC5892128; doi:10.1039/c7sc04091a)
Supplement: Supplementary file 1 [file SC-009-C7SC04091A-s001.pdf]

Electronic Supplementary Material (ESI) for Chemical Science.
This journal is © The Royal Society of Chemistry 2018

# A New Twist in the Photophysics of the GFP Chromophore: A Volume-Conserving Molecular Torsion Couple

Jamie Conyard[1], Ismael A. Heisler[1], Yohan Chan[1], Philip C. Bulman Page[1], Stephen R. Meech[1]* and Lluís Blancafort[2]*

[1]*School of Chemistry, University of East Anglia, Norwich Research Park, Norwich NR4 7TJ, UK and* [2]*Institut de Química Computacional i Catàlisi and Departament de Química, Facultat de Ciències, Universitat de Girona, C/ M. A. Capmany 69, 17003 Girona, SPAIN*

KEYWORDS (Ultrafast; Photoswitch; green fluorescent protein; photophysics; excited state; minimum energy pathway; isomerization).

**Abstract**

The simple structure of the chromophore of the green fluorescent protein (GFP), a phenol and an imidazolone ring linked by a methyne bridge, supports an exceptionally diverse range of excited state phenomena. Here we describe experimentally and theoretically the photochemistry of a novel sterically crowded nonplanar derivative of the GFP chromophore. It undergoes an excited state isomerization reaction accompanied by an exceptionally fast (sub 100 fs) excited state decay. The decay dynamics are essentially independent of solvent polarity and viscosity. Excited state structural dynamics are probed by high level quantum chemical calculations revealing that the fast decay is due to a conical intersection characterized by a twist of the rings and pyramidalization of the methyne bridge carbon. The intersection can be accessed without a barrier from the pre-twisted Franck-Condon structure, and the lack of viscosity dependence is due to the fact that the rings twist in the same direction, giving rise to a volume-conserving decay coordinate. Moreover, the rotation of the phenyl, methyl and imidazolone groups are coupled in the sterically crowded structure, with the methyl group translating the rotation of one ring to the next. As a consequence, the excited state dynamics can be viewed as a torsional couple, where the absorbed photon energy leads to conversion of the out-of-plane orientation from one ring to the other in a volume conserving fashion. A similar modification of the range of methyne dyes may provide a new family of devices for molecular machines, specifically torsional couples.

Authors for correspondence* s.meech@uea.ac.uk; lluis.blancafort@udg.edu

## INTRODUCTION

Photoswitches play important roles in biology. Examples include the primary step in the vision pigment rhodopsin, bacterial phototaxis stimulated by the photoactive yellow protein and fluorescent protein (FP) photochromism used in super-resolution microscopy.[1-3] These efficient protein based photoswitches inspired the design of diverse molecular photoswitches, which power a variety of nano- and micro-scale phenomena.[4-9] In the following we investigate the photophysics of a novel sterically crowded variant of the chromophore of the green fluorescent protein (GFP), by means of ultrafast spectroscopy and high-level quantum chemical calculations. This bridge methylated derivative (see Scheme 1a,b) shows an exceptionally fast excited state decay which is almost independent of solvent viscosity. In contrast to the native GFP chromophore, whose decay is calculated to be governed by ring rotation, the presently calculated excited state structural evolution suggests that the methylated derivative follows a barrierless, volume conserving coordinate composed of ring rotation and pyramidalization of the central carbon. We further show that this arises from a nonplanar form of the chromophore analogous to that found in some photoactivated FPs.[10]

The FP family is established as one of the most important tools in bioimaging and cell biology.[11-14] A significant and intriguing feature is the wide range of photophysical phenomena exhibited by the covalently bound chromophore common to most of them (Figure 1).[15] In recent years this diversity afforded FPs a range of applications beyond bioimaging. The chromophore exhibits, depending on its environment: photochromism, critical to applications in super-resolution imaging[3, 16, 17]; excited state proton transfer[18-20]; photo-isomerization[21]; intermolecular photochemical reaction (exploited in “optical highlighter” proteins)[22-24]; electron transfer (generating reactive oxygen species leading to photo-stimulated cell death)[25]. In an effort to understand this diversity, the synthetic analogue of the FP chromophore (4’-hydroxybenzylidene-2,3-dimethylimidazolinone, p-HBDI, Figure 1) has been studied intensely, through both experiment and quantum chemical calculation.[26-38] The extended delocalisation leads to a visible absorbing chromophore which is approximately planar in its

electronic ground state and adopts the cis (Z) isomer (although it may be twisted or even trans (E) in the protein environment). The photophysics of p-HBDI are dominated by ultrafast radiationless decay and isomerization.[21, 28, 39, 40] Quantum chemical calculations suggest that excited state population decay occurs at a conical intersection (CI) reached by an approximately 90° rotation about the $\tau$ or $\phi$ twisting coordinate (Scheme 1).[34, 35, 37, 41-43] Such large scale molecular motions are opposed by solvent friction, and thus predict a solvent viscosity dependence, which is not observed experimentally. Consequently other radiationless decay coordinates which displace less solvent, notably "hula-twist" or pyramidalization at the central bridging methyne group, have been considered.[42, 44, 45]

These observations led to efforts to control motion along coordinates involving the bridging bonds. Chou and co-workers made a 'locked HBDI' with a 5 membered ring restraining the $\phi$ coordinate.[46] The excited state lifetime was only slightly extended compared to p-HBDI. Remarkably, when the intramolecular H-bonded ortho hydroxy derivative was studied, the fluorescence lifetime increased by three orders of magnitude, to the nanosecond range. In contrast o-HBDI itself has a more modest lifetime enhancement of about 1 order of magnitude. Thus, both $\tau$ and $\phi$ coordinates should be constrained to recover the high quantum yield associated with imaging FPs, consistent with earlier studies of a boron coordination complex.[47] Very recently other routes to $\tau$ locked HBDI-like structures were reported. Although no direct comparison was made with HBDI, the excited state decay remained sub-picosecond.[48, 49]

These data suggest a key role for the bridging carbon. In this work we synthesized the bridge methylated derivative of HBDI (1-(4-hydroxyphenyl)ethylidene-2,3-dimethylimidazolinone, **I**, Scheme 1) which yields a sterically crowded nonplanar structure where neither bond is rigidly constrained. Steric crowding has previously been seen to accelerate the excited state decay of stilbene derivatives,[50] and a similar acceleration has also been recently observed when the bridge carbon of a model bilirubin chromophore, structurally related to HBDI, is methylated[51]. The synthesis of **I**

affords us the opportunity to investigate in detail the excited state dynamics of a nonplanar GFP chromophore. It has been reported from a systematic study of the structures of GFP-like proteins that the chromophore is often nonplanar, a phenomenon ascribed to the steric effect of the surrounding matrix.[52] It was also reported that a variation in the size of the Y145 residue adjacent to the chromophore, could have a controlling influence on the fluorescence quantum yield, again probably due to a steric effect.[53] A more extreme perturbation of chromophore structure by the host protein is in the generation of the less common trans form, which is then often significantly distorted from a planar structure, and exhibits both weak fluorescence and photochromism.[10, 16, 54] The latter point is central to the application of photoconvertible proteins such as dronpa in super-resolution fluorescence bioimaging. These factors all suggest that an investigation of the photophysics of a nonplanar chromophore outside the protein matrix may be important in assessing the role of nonplanar geometries in the photophysics of GFP-like proteins.

Thus, we present a detailed experimental and theoretical study of the excited state chemistry of **I**. The decay is ultrafast, even compared to the sub-picosecond decay of p-HBDI, and is only a very weak function of the environment. These observations are explained through high level calculations combining time-dependent density functional theory (TD-DFT) using the CAM-B3LYP functional with the complete active space second-order perturbation (CASPT2) method. The ultrafast decay arises from a unique excited state structural reorganization, which reveals the sterically crowded **I** as a volume-conserving molecular "torsion couple". The molecular motions required are driven by bond inversion promoting phenol ring planarization coupled to imidazole ring rotation through the methyl group. In the ground state the rings are already twisted, and after excitation they rotate in the same direction, so that the decay to the ground state can occur with minimal changes in the volume. The generalization of this mechanism may explain some of the results outlined above, such as the short lifetimes of some 'locked' derivatives.

## RESULTS AND DISCUSSION

### Electronic Structure and Photochemistry

The absorption spectra of the neutral protonated form of **I** are shown in Figure 1a, in a series of alcohol solvents of different viscosity and polarity. The absorption is a single asymmetric band with maximum near 360 nm, and is only a weak function of solvent. This is slightly to the blue of p-HBDI and has a smaller extinction coefficient of ca 14000 $M^{-1}cm^{-1}$ compared to 32000 $M^{-1}cm^{-1}$. NMR data (electronic supplementary information 1, ESI 1) show that **I** is synthesized in the cis (*Z*) and trans (*E*) isomers in the ratio 9:1. We refer to these isomers as *Z*-**I** and *E*-**I**, respectively. The absence of a strongly bimodal line shape suggests the isomers have similar spectra.

Photochemical measurements show that irradiation into $S_1$ with 365±20 nm light converts Z to E in an analogous fashion to p-HBDI,[21] confirming that Z and E isomers have similar spectra although E has an additional shoulder below 300 nm (Fig. 2b); that no additional photoproducts are formed was confirmed by NMR. Photoconversion kinetics were measured and analysed to recover yields for the Z→E and E→Z reactions (Figure 1). Analysis of these data (Table 1 and ESI 4) shows that the Z→ E photoconversion yield is ca 4±2%, but significantly larger for the reverse reaction at 25±10%. The ground state E to Z relaxation rates in water and methanol are 3 and 7 times greater respectively than for p-HBDI (see ESI 4), which corresponds to differences in the activation energy of 0.7 - 1.2 kcal·$mol^{-1}$ at room temperature. For comparison, the calculated gas-phase barriers for the thermal E→Z pathway (MS-CASPT2 energies on CASSCF geometries, see ESI 3) are 44.5 and 44.0 kcal/mol for p-HBDI and **I**, respectively (Figure 2). This corresponds to a 2.5 times faster relaxation rate for **I**, in reasonable agreement with the experimental data. However, the recovery time of p-HBDI and **I** in the aprotic solvent acetonitrile is very long (ESI 4), suggesting a significant solvent dependence of the E→Z reaction rate. A similar observation has been made for p-HBDI.[21] Such medium effects will be important in understanding relaxation kinetics in photochromic proteins and will be studied in a more extensive range of solvents and solvent mixtures.

The fluorescence of **I** is very weak, but the spectra have an approximately mirror image relationship to the absorption, and are a weak function of the solvent (Figure 1a). All the alcohol solvents studied show a maximum **I***  emission wavelength around 427 nm although the diol, ethylene glycol, is slightly blue-shifted. In this paper we focus throughout on the neutral protonated chromophore, which is often the most photochemically active form of the FP chromophores, but very similar experimental results were obtained for the deprotonated chromophore in basic solvents (ESI 5).

The calculated minimum energy ground state structure of **I** at the MP2/cc-pvtz level has the *Z* configuration around the C5-C7' double bond and is markedly non-planar ($C_1$ symmetry), with $\phi$ = 29.8° and $\tau$ = 2.1° (Figure 2). This is traced to the steric interaction between the methyne methyl substituent and the two rings, which prevents formation of a stable planar minimum. The departure from planarity gives rise to the observed blue-shift and reduced oscillator strength of *Z*-**I** compared to the planar p-HBDI. The barrier for rotation around $\phi$, which goes through a planar transition structure and leads to a mirror inverted structure where the rings are rotated in opposite directions, is 0.6 kcal·mol$^{-1}$ (Figure 2). The *E*-**I** minimum is 0.7 kcal·mol$^{-1}$ higher in energy than *Z*-**I** and is slightly more twisted, with $\phi$ = 44.5° and $\tau$ = −176.7°. The calculated energy difference predicts that approximately 69 % of the ground-state population will correspond to the *Z* form at 300 K, in line with the 9:1 ratio seen in NMR (ESI 1).

The ground-state energy profile for the rotation of the methyl group is particularly significant as it illustrates the function of the proposed torsional couple discussed below. The rotation of the methyl group ($\rho$ coordinate, Scheme 1) is accompanied by rotation of the phenoxy group, and the calculated barrier for methyl rotation in the *Z*-**I** form is 0.7 kcal·mol$^{-1}$ (see Figure ESI3). For this barrier we estimate room temperature methyl group rotation to occur on a timescale of 0.3 – 3 ps (assuming a pre-exponent of $10^{12}$ – $10^{13}$ s$^{-1}$ [55]) which is slow on the 100 fs timescale of the excited state dynamics (see below) but fast on the NMR timescale (hence only a single NMR peak is seen for the methyl group).

The vertical absorption energies were calculated with MS-CASPT2 and TD-CAM-B3LYP at the MP2/cc-pvtz optimized ground-state geometries, assuming a gas-phase environment (which is appropriate given the weak solvent effect, Figure 1). Table 2 shows the calculated vertical excitation energies for *Z*-**I** below 5 eV, while those for *E*-**I** are reported in ESI 3. At the MS-CASPT2 level, the $S_1$ excitation (*Z*-**I)** is 3.50 eV (354 nm), in good agreement with the experimental data (360 nm). The excitation corresponds to the HOMO (H) ⟶LUMO (L) transition and is allowed (oscillator strength 0.31); the most relevant orbitals at the minimum *Z*-**I** structure are shown in Figure 3, illustrating the methyne bridge bond alternation on excitation. The $S_1$ energy at the TD-CAM-B3LYP level is somewhat higher than observed (3.71 eV, 328 nm). The next two states are an ($n_O$,$\pi$*) state, where the excitation comes from the oxygen lone pair of the imidazolone ring, and a state localized on the phenoxy ring analogous to the benzene $B_{2u}$ state. These appear at 4.35 and 4.51 eV at the MS-CASPT2 level, but have low oscillator strength. The calculated $S_1$ excitation energy and oscillator strength for *E*-**I** are 3.40 eV (365 nm) and 0.13, somewhat lower than for the *Z*-**I** minimum, consistent with Figure 1b.

**Ultrafast Fluorescence**

Figure 4 shows time resolved fluorescence of **I** measured with sub 50 fs time-resolution fluorescence up-conversion,[56] as a function of emission wavelength and solvent. The analysis in terms of a sum of two exponential decay terms, which accurately fits all data, is presented in Table 3. In all cases the decay is exceptionally fast, being dominated (ca 90 %) by a component of 70 ± 20 fs. The second component decays on a slightly slower timescale, but always faster than 400 fs. We obtained the same result for the anion $\mathbf{I}^-$, although the decay times are slightly longer, and the longer lived component has a higher weight (ESI 5).

These data show that the excited state decay of **I** is approximately a factor of four faster than that of p-HBDI, which is already sub-picosecond(Figure 4)[39]. The measurements were made in a chemically similar series of alcohol solvents, in which viscosity varied by a factor of 30. The effect on the mean

excited state lifetime is at most a factor of 1.5, showing that the coordinate leading to radiationless decay is essentially independent of viscosity (Figure 4b), which is different to the case for sterically hindered stilbenes.[50] Inspection of Table 3 shows that even this small viscosity dependence is mainly carried by the lower amplitude 'slow' component. Further, no dependence on solvent polarity was observed. Figure 4c shows that the emission wavelength dependence is weak, although on the red edge of the spectrum the longer component has an increased amplitude, which is reflected in a small but reproducible wavelength dependence in the mean relaxation time (Table 3). The absence of a risetime in the emission at any wavelength shows that the fluorescent state is formed within the time resolution of the measurement. The near wavelength independence shows that neither the shape nor the energy of the fluorescence spectrum is evolving significantly in time, as was also observed for p-HBDI.[57]

The observed ultrafast viscosity and polarity independent decay, combined with a time independent spectrum is difficult to reconcile with the most well-established mechanisms for excited state isomerization.  These typically invoke diffusive motion along a reaction coordinate (for example a single bond rotation) in the excited electronic state to access a CI, a mechanism which is expected to show a strong viscosity effect.[58-60] Such models have been successfully applied to picosecond time scale excited state reactions. More recently a number of sub-picosecond excited state isomerization reactions have been reported, where ultrafast relaxation suggests reaction coordinates which do not displace large solvent volumes, and are therefore less sensitive to solvent viscosity. Examples include ultrafast isomerization in cis-stilbene and rhodopsin, in which hydrogen out-of-plane (HOOP) motion plays a key role in driving the reaction to the CI on an ultrafast timescale.[61, 62] Such measurements stimulated our synthesis of **I**, where simple kinematics suggested that methylation might slow the reaction relative to p-HBDI. The observed acceleration is therefore inconsistent in principle with HOOP modes playing a key role in the radiationless decay of **I**. A similar low volume coordinate which has been invoked in sub-picosecond excited state reactions is pyramidalization at an ethylenic

carbon atom[63]. However, there is again no reason to predict that motion along such a volume conserving coordinate would be markedly accelerated by methylation.

To better understand the origin of the remarkably fast decay in **I** we calculated the excited state minimum energy path (MEP), which reveals that the acceleration arises from the sterically crowded non planar structure, and further, that this gives rise to a highly cooperative excited state structural reorganization, resulting in transfer of torsion between the two rings. The MEP calculations for the *Z*-**I** and *E*-**I** isomers are summarized in Figure 5, where displacements are measured in atomic units, a.u. (bohr·amu$^{1/2}$). The paths (Figure 5a) were obtained with TD-CAMB3LYP optimizations, and the energies refined with MS-CASPT2 (see Computational Details). Both isomers have similar, bimodal MEPs characterized by an initial steep decay (approximately 0 - 1 a. u. displacement) associated to bond inversion, followed by an extended flat region (a plateau) where the main motion is ring rotation. Note that the initial part of the path (0 - 5 a. u.) is plotted in a different scale for clarity. The path leads without a barrier to an $S_1/S_0$ CI that lies 1 eV below the vertical excitation (2.45 and 2.33 eV for *Z*-**I** and *E*-**I**, respectively). Representative structures along the decay paths are shown in Figure 5b, namely the FC and CI structures for both isomers and the structures at the beginning of the plateau (1.2 a.u. displacement), labeled Plat. The structural changes are further detailed in ESI 3. We center our discussion on the *Z* isomer (see the angle definitions in Scheme 1), and for comparison we provide also the data for the *E* form, which follows a similar course.

From the structural point of view (Figure 5b), the initial steep decay phase is characterized by inversion of the central bonds; for *Z*-**I,** the $C_7$'-$C_5$ bond, which is a double bond in the ground state (Scheme 1), stretches from 1.37 to 1.42 Å at *Z*-Plat, while the $C_{1'}$-$C_{7'}$ bond, which has single bond character in $S_0$, is shortened from 1.47 Å to 1.42 Å (see Figure S4 for the evolution of the distances along the whole path). This is in line with the character of the orbitals involved in the excitation, since the occupied orbital is bonding along the $C_7$'-$C_5$ bond and antibonding along $C_{1'}$-$C_{7'}$, and the virtual orbital has the opposite character (Figure 3). At the beginning of the plateau, the calculated $S_1/S_0$ energy gap is approximately 2.8 eV and decreases slowly. The calculated value is in agreement

with the measured emission maximum of 2.9 eV, and suggests that the fluorescence comes mainly from the fraction of molecules that resides in this plateau region. To reach the plateau, **I** only undergoes changes in the bond lengths, which is consistent with the absence of a rise time in fluorescence and the mirror image relationship between the absorption and emission spectra. The remaining part of the decay path is characterized by rotation of the two rings. For the major *Z*-**I** isomer, there is a continuous decrease of the oscillator strength along the path (see figure S5). This supports the idea that most of the fluorescence comes from the molecules in the initial plateau region. In contrast, *E*-**I** has a lower oscillator strength that almost does not change along the path.

The bond rotation along the path occurs in response to the inversion in bond character (see Figures 5b and ESI3). The phenol ring, which is initially rotated out-of-plane, becomes co-planar with the central $C_5$-$C_{7'}$-$C_{1'}$ unit, *i.e.* $\phi$ decreases from approximately 30° to nearly 0° and further to -17°. In turn, the imidazolone ring twists out of the plane until it becomes perpendicular to the central plane, *i.e.* $\tau$ increases from 2.1°, to reach a final value of approximately 90° at the CI (see Figure S6). The CI structure is consistent with previous studies for the neutral and anionic forms of HBDI, where the minimum energy CI is found at $\phi$ = -30 - 25° and $\tau$ = 75 - 103°.[35, 37, 43] The MEP is also similar to the one calculated for the sterically crowded bilirubin model chromophore, although there the decay, measured by transient absorption, takes place on a slower time scale.[51] Significantly, in our case the methyl group (dihedral angle $\rho$ Scheme 1) rotates simultaneously with the phenol and imidazole rings (see Figure S6). This behavior is also seen for the *E*-**I** isomer and demonstrates the idea that the ring rotations are coupled by the methyl group.

The importance of ring torsion for the radiationless decay may seem at odds with the absence of a viscosity effect. Large scale structural changes should be opposed by solvent friction, which has not been observed (Figure 4b). However, this can be readily understood considering that the two rings are rotating in the same direction, which results in small volume changes during the decay. This can be quantified in terms of a 'flapping' angle $\gamma$ (see Scheme 1), which is an effective measure of the

relative motion of the two rings. Along the decay MEP (see Figure S7a), there is only a small increase of $\gamma$ for *Z*-**I** (from 27° at *Z*-FC to 51° at *Z*-CI), whereas $\gamma$ stays almost unchanged at values of 45°-50° for *E*-**I**. From this perspective, the main coordinate that drives the decay is the pyramidalization of the central, $C_{7'}$ carbon (see Figure S7 for a quantitative discussion of the pyramidalization).

Our analysis of the flapping and pyramidalization angles supports a mechanistic picture where the CI is characterized, both for *Z* and *E* forms, by a flapping angle of approximately 50° between the two rings and a pyramidalized central carbon. Due to the steric crowding, the ground state structure is already significantly pretwisted. This probably accelerates the decay compared to the unsubstituted structure, as suggested for the related chromophores[29, 51]. More importantly, it reduces the changes in volume required to access the CI. This is illustrated in Figure 6, where the FC and CI structures are superimposed for both isomers. The images show that for both isomers, one of the main differences between the FC and CI structures is the position of the methyl group. The importance of this volume-conserving pyramidalization coordinate for the decay is thus consistent with the ultrafast decay and the lack of a viscosity effect.

The passage through the CI can lead to double bond isomerization if the direction of imidazolone rotation is maintained after the decay. However, the small changes required to access the CI and the larger slope of the ground state surface at the CI compared to the excited state, suggest that return to the reactant configuration will be favoured. This is consistent with the small isomerization quantum yields, which are estimated at about 4 and 25 % for *Z*-**I** and *E*-**I**, respectively. Remarkably, the two isomers have significantly different yields, in spite of the structural similarity between the *Z*-**CI** and *E*-**CI** structures. To explain this one must consider that the two CIs are probably part of an extended seam of intersection,[64, 65] similar to the one characterized in detail for HBDI,[43] and the different yields may be due to the dynamics of the decay at the seam.†

The excited-state dynamics of a molecular system depend crucially on how the excitation energy is converted into nuclear motion. In the HBDI family of rotors, as well as in others such as retinal,[66]

diazobenzene[67] or fulvene-based systems,[68, 69] the modes initially excited are bond stretching modes associated with bond inversion (Figure 5). The excited-state lifetime then depends on how the energy flows from these to the torsional modes, *i.e.* on intramolecular vibrational energy redistribution (IVR). There are two IVR-related features that distinguish **I** from other rotors, which explain its extremely fast dynamics and suggest its role as a photoswitch. The first one is coupling between the orientations of the two rings provided by the methyl group. Initially the main torque is applied on the phenyl group, which is driven towards planarity by bond inversion, and the coupling through the methyl group translates this rotational impulse to the imidazole ring, which then has to twist to reach the conical intersection. This additional driving force distinguishes **I** from other photoswitches such as the azobenzenes.[8] Secondly, **I** is already twisted in its ground-state structure, and this favours efficient IVR from the stretching to the torsional modes.

This result is more general than in the present specific case of **I**. The GFP chromophore is an example of a wider family of mono-methyne dyes, many of which exhibit strong absorption and weak fluorescence and undergo excited state structure change.[70] It is likely that bridge methylation in these cases would also lead to steric crowding, a nonplanar ground state and thus the excited state torsional coupling, as illustrated in Figure 5. The advantage of access to this broad family of dyes is the variety of aromatic rings available, which then offers a range of synthetic targets, providing synthetic chemists an opportunity to produce torsional couples with higher isomerization yields than **I** and larger angular displacements between the rings. Further, such a range of nonplanar methyne bridged ground states could be coupled to different molecular and supramolecular structures, to exploit the structure change. Of course the exploitation of such a torsional couple depends on the synthesis of derivatives with larger photochemical cross sections and bigger displacements.

Finally, we discuss the role of non-planarity in relation to the chromophore of GFP-like proteins, which arises due to steric crowding in the protein matrix. Our observation of an accelerated excited state decay in the nonplanar **I** compared to HBDI (Figure 3) is at first sight consistent with the observation of weak fluorescence from highly nonplanar trans forms of the chromophore in GFP-like

proteins. [71] However the relationship between planarity and quantum yield is generally not a simple one, with fluorescence being observed for proteins with a wide range of angles about both $\tau$ and $\phi$ twists, for both cis and trans chromophores;[52] as far as we are aware there is no systematic study of fluorescence yield as a function of geometry. Of perhaps greater significance is our characterization of a volume conserving pyramidalization at the bridging carbon in the radiationless decay coordinate of the nonplanar **I**. It is not straightforward to see how a simple steric effect in the protein might suppress such a motion, and thereby enhance the fluorescence quantum yield to the very high values characteristic of GFPs used in bioimaging. To gain further insight into this point, we will reassess the decay of the unsubstituted HBDI chromophore in future work. However, in addition to the protein modifying the steric environment of the chromophore it also alters the electronic structure, through intermolecular interactions and through changes to the electrostatic environment. These changes can result in significant 'protein shifts' in the energy of the electronic transitions.[72] It is possible that such interactions, rather than steric effects, play a role in determining the fluorescence quantum yield. This may occur if such interactions either modify the position of conical intersections, or otherwise steer the excited state structural evolution away from them. In this connection, the recent characterization of a key role for the electrostatic environment of the chromophore in controlling photoprotein fluorescence yield may be significant.[73]

**CONCLUSIONS**

We have synthesized a novel nonplanar form of the GFP chromophore, **I,** and investigated its photophysics. A number of GFP chromophores have been previously synthesized with a view to stabilizing the planar geometry and enhancing fluorescence. In contrast **I** is sterically crowded and has a nonplanar ground state, which nevertheless undergoes an extremely fast excited state isomerization. Ultrafast fluorescence shows that the excited state decay is greatly accelerated compared to planar p-HBDI. Quantum chemical calculations reveal a barrierless MEP to an $S_1/S_0$ intersection where radiationless decay and *Z/E* isomerization occurs. The calculations reveal a

plateau in the early part of the relaxation, where the excited state resides for its ca 70 fs lifetime. As a consequence of the pre-twisted ground-state geometry, the CI can be reached by a volume conserving coordinate composed of torsion of the rings in the same direction and simultaneous pyramidalization of the methyne bridge carbon, consistent with the observed negligible solvent viscosity effect. Significantly, the MEP also reveals strong coupling between the torsional angle of the two rings. Electronic excitation leads to bond inversion in the bridging methyne, which drives the phenol ring towards planarity. The orientation of the phenyl ring is coupled to the imidazolone ring orientation via the methyl substituent, causing it to be driven out-of-plane. This can be viewed as a light driven molecular torsion couple. Importantly, the excited state evolution described is unlikely to be restricted to **I**. There is a large family of related monomethyne dyes which share a similar bridging motif and electronic structure to p-HBDI.[74] This promises a range of possibilities to both modify MEPs to optimise isomerization yield and to design sites which allow the proposed light driven torsion couple to be incorporated into molecular machines.

**Experimental Methods**. The synthesis and characterization of **I** are described in detail in ESI 1. The synthesis was based on the methods described by Burgess and Wu [75] for the preparation of the imidazolone moiety, and Hsu et al.[46] for the coupling between the ketone and the imidazolone compounds.

Time resolved fluorescence measurements were made with a previously described up-conversion spectrometer (See ESI 2).[56] The excitation wavelength was 400 nm and emission at wavelengths from 470 nm to 550 nm was up-converted with 800 nm pulses. The sample was contained in a 1 mm pathlength cell.

**Computational Details.** Ground-state optimizations of the neutral form of **I** in the gas phase were carried out at the MP2/cc-pvtz level of theory. The excited state decay path from the Franck-Condon (FC) region to the CI was mapped with a series of constrained optimizations where points on the potential energy surface are optimized on a hypersphere with a fixed radius centred on an initial

pivot point.[76] The path was obtained using the optimized point of every calculation as pivot point for the following step, and the mass-weighted displacements are given in atomic units (a.u.), *i.e.* bohr·amu$^{1/2}$. For optimizations we used TD-CAM-B3LYP/6-311g** and the Gaussian program.[77] CASSCF/6-311g** excited-state optimizations were discarded because this method fails to give the right state order near the FC region,[35] and it overestimates the $S_1$-$S_0$ energy gap in the vicinity of the CI. *Z*-CI and *E*-CI are the last points of each decay path, where the TD-CAM-B3LYP optimizations fail to converge. The energies along the paths were recomputed at the MS-CASPT2/ANO-S level of theory with Molcas[78] to provide a uniform picture of the reaction paths at the multireference, dynamically correlated level. Further computational details are provided in the SI.

Footnote:

†The small differences in the shape of the MEPs near the CI visible in Figure 5a, where the MEP for *Z*-**I** appears to have a minimum close to the CI, are due to the failure to converge the last step of the TD-CAM-B3LYP MEP optimizations and not to differences in the seam topography at the CI. MS-CASPT2 calculations along the gradient difference coordinate showed that the seam has very similar topography at *Z*-CI and *E*-CI.

**Acknowledgements**

LB acknowledges financial support from Spanish Ministerio de Economía y Competitividad (UNGI10-4E-801, CTQ2015-69363-P), Departament d'Innovació, Universitats i Empresa (DIUE) from Generalitat de Catalunya (2014SGR-1202), and computational time from  Consorci de Serveis Universitaris de Catalunya. SRM is grateful to EPSRC for financial support (EP/M001997/01)

**Electronic Supporting Information (ESI) available**

Synthetic methods and characterization; Fluorescence up-conversion data; Additional computational details; Cartesian coordinates of key structures; Photochemical isomerization data; and Data for the anion of **I**.

TABLES

**Table 1**. Rate of photoconversion (dominant Z → PSS (photostationary state) and rate of dark relaxation PSS → Z for **I**. All photoconversion measurements were made under identical conditions (excitation at 365nm with irradiance of ca. 2 mW $cm^{-2}$, and a sample absorbance of 0.14 at 365nm). Relaxation measurements were conducted on the same samples immediately after stopping irradiation. For further analysis and cross section calculation see SI 4

| **Solvent** | $\tau_{Z \rightarrow PSS}$ / s | $\tau_{PSS \rightarrow Z}$ / s |
|---|---|---|
| **Methanol** | 3.56 | 497.9 |
| **Ethylene Glycol** | 6.65 | 551.1 |
| **Water** | 4.20 | 12.0 |
| **Acetonitrile** | 2.95 | 2309.6 |

**Table 2.** Vertical excitation spectrum (lowest four states) of the MP2/cc-pvtz optimized *Z*-**I** minimum calculated with MS-CASPT2 and TD-CAM-B3LYP. Energies in eV and oscillator strength in brackets

| State | MS-CASPT2/ANO-S[a] | | TD-CAM-B3LYP/cc-pvtz | |
|---|---|---|---|---|
| | $E_{ex}$ [eV][b] | Character[c] | $E_{ex}$ [eV][b] | Character[c] |
| $S_1$ | 3.50 (0.312) | H→L ($\pi$,$\pi$*)[c] | 3.71 (0.682) | H→L ($\pi$,$\pi$*) |
| $S_2$ | 4.35 (0.005) | ($n_O$,$\pi$*) | 4.04 (0.037) | mixed (n,$\pi$*)/$B_{2u}$ |
| $S_3$ | 4.51 (0.0002) | H→L+1, H-2→L ($B_{2u}$-like) | 4.71 (0.064) | H-1→L ($\pi$,$\pi$*) |
| $S_4$ | 4.73 (0.071)[d] | H-1→L ($\pi$,$\pi$*) | 4.77 (0.004) | mixed (n,$\pi$*) / $B_{2u}$ |

[a]Active space (16,14), wave function averaged over five states. [b]Oscillator strength in brackets. [c]See Figure 1c for H and L orbitals (HOMO and LUMO). See Figure S2 in the ESI for the other orbitals and the wave function configuration coefficients. [d]Obtained from calculation over six averaged states.

**Table 3**. Time constants ($\tau$) and weighting factors ($A$) at a given emission wavelength ($\lambda$) for a fit of the fluorescence decay of **I** to a sum of two exponential terms. The mean lifetime, $<\tau>$ is also calculated.

| Solvent | $\lambda$/nm | $\tau_1$/fs, $A_1$ | $\tau_2$/fs, $A_2$ | $<\tau>$/fs |
|---|---|---|---|---|
| **Methanol** | 470 | 70, 1.0 | - | 70 |
| **Methanol** | 500 | 50, 0.91 | 190, 0.09 | 70 |
| **Methanol** | 550 | 80, 0.95 | 390, 0.05 | 90 |
| **Propan-1-ol** | 470 | 60, 0.93 | 200, 0.07 | 70 |
| **Butan-1-ol** | 470 | 70, 0.98 | 360, 0.02 | 80 |
| **Octan-1-ol** | 470 | 70, 0.98 | 490, 0.08 | 80 |
| **Ethylene Glycol** | 470 | 60, 0.95 | 410, 0.05 | 80 |

## References


1. M. A. Van der Horst and K. J. Hellingwerf, *Acc. Chem. Res.*, 2004, 37, 13-20.
2. A. Wand, R. Rozin, T. Eliash, K.-H. Jung, M. Sheves and S. Ruhman, *J. Am. Chem. Soc.*, 2011, 133, 20922-20932.
3. F. Lavoie-Cardinal, N. A. Jensen, V. Westphal, A. C. Stiel, A. Chmyrov, J. Bierwagen, I. Testa, S. Jakobs and S. W. Hell, *Chemphyschem*, 2014, 15, 655-663.
4. D. J. van Dijken, P. Kovaříček, S. P. Ihrig and S. Hecht, *J. Am. Chem. Soc.*, 2015, 137, 14982-14991.
5. C. Poloni, M. C. A. Stuart, P. van der Meulen, W. Szymanski and B. L. Feringa, *Chemical Science*, 2015, 6, 7311-7318.
6. J. C. M. Kistemaker, P. Štacko, J. Visser and B. L. Feringa, *Nat Chem*, 2015, 7, 890-896.
7. J. Briand, O. Bram, J. Rehault, J. Leonard, A. Cannizzo, M. Chergui, V. Zanirato, M. Olivucci, J. Helbing and S. Haacke, *PCCP*, 2010, 12, 3178-3187.
8. M. Irie, *Chem. Rev.*, 2000, 100, 1683-1683.
9. T. Kudernac, N. Ruangsupapichat, M. Parschau, B. Macia, N. Katsonis, S. R. Harutyunyan, K.-H. Ernst and B. L. Feringa, *Nature*, 2011, 479, 208-211.
10. M. Andresen, A. C. Stiel, S. Trowitzsch, G. Weber, C. Eggeling, M. C. Wahl, S. W. Hell and S. Jakobs, *Proceedings of the National Academy of Sciences of the United States of America*, 2007, 104, 13005-13009.
11. A. Miyawaki, *Dev. Cell*, 2003, 4, 295-305.
12. M. V. Matz, K. A. Lukyanov and S. A. Lukyanov, *Bioessays*, 2002, 24, 953-959.
13. G. U. Nienhaus, *Angewandte Chemie-International Edition*, 2008, 47, 8992-8994.
14. R. Y. Tsien, *Annu. Rev. Biochem.*, 1998, 67, 509-544.
15. S. R. Meech, *Chem. Soc. Rev.*, 2009, 38, 2922-2934.
16. H. Mizuno, T. K. Mal, M. Walchli, A. Kikuchi, T. Fukano, R. Ando, J. Jeyakanthan, J. Taka, Y. Shiro, M. Ikura and A. Miyawaki, *Proceedings of the National Academy of Sciences of the United States of America*, 2008, 105, 9227-9232.
17. P. Dedecker, J. Hotta, R. Ando, A. Miyawaki, Y. Engelborghs and J. Hofkens, *Biophys. J.*, 2006, 91, L45-L47.
18. M. Chattoraj, B. A. King, G. U. Bublitz and S. G. Boxer, *Proceedings of the National Academy of Sciences of the United States of America*, 1996, 93, 8362-8367.
19. D. Stoner-Ma, A. A. Jaye, P. Matousek, M. Towrie, S. R. Meech and P. J. Tonge, *J. Am. Chem. Soc.*, 2005, 127, 2864-2865.
20. O. Vendrell, R. Gelabert, M. Moreno and J. M. Lluch, *J. Am. Chem. Soc.*, 2006, 128, 3564-3574.
21. V. Voliani, R. Bizzarri, R. Nifosi, S. Abbruzzetti, E. Grandi, C. Viappiani and F. Beltram, *J. Phys. Chem. B*, 2008, 112, 10714-10722.
22. I. Hayashi, H. Mizuno, K. I. Tong, T. Furuta, F. Tanaka, M. Yoshimura, A. Miyawaki and M. Ikura, *J. Mol. Biol.*, 2007, 372, 918-926.
23. T. Kogure, S. Karasawa, T. Araki, K. Saito, M. Kinjo and A. Miyawaki, *Nat. Biotechnol.*, 2006, 24, 577-581.
24. J. Tay, M. A. Parkes, K. Addison, Y. Chan, L. Zhang, H. C. Hailes, P. C. Bulman Page, S. R. Meech, L. Blancafort and H. H. Fielding, *The Journal of Physical Chemistry Letters*, 2017, DOI: 10.1021/acs.jpclett.7b00174, 765-771.
25. M. E. Bulina, D. M. Chudakov, O. V. Britanova, Y. G. Yanushevich, D. B. Staroverov, T. V. Chepurnykh, E. M. Merzlyak, M. A. Shkrob, S. Lukyanov and K. A. Lukyanov, *Nat. Biotechnol.*, 2006, 24, 95-99.

26. C. L. Walker, K. A. Lukyanov, I. V. Yampolsky, A. S. Mishin, A. S. Bommarius, A. M. Duraj-Thatte, B. Azizi, L. M. Tolbert and K. M. Solntsev, *Curr. Opin. Chem. Biol.*, 2015, 27, 64-74.
27. L. M. Tolbert, A. Baldridge, J. Kowalik and K. M. Solntsev, *Acc. Chem. Res.*, 2012, 45, 171-181.
28. K. Addison, J. Conyard, T. Dixon, P. C. B. Page, K. M. Solntsev and S. R. Meech, *Journal of Physical Chemistry Letters*, 2012, 3, 2298-2302.
29. J. Conyard, M. Kondo, I. A. Heisler, G. Jones, A. Baldridge, L. M. Tolbert, K. M. Solntsev and S. R. Meech, *J. Phys. Chem. B*, 2011, 115, 1571-1576.
30. J. Dong, K. M. Solntsev and L. M. Tolbert, *J. Am. Chem. Soc.*, 2009, 131, 662-670.
31. J. Dong, K. M. Solntsev, O. Poizat and L. M. Tolbert, *J. Am. Chem. Soc.*, 2007, 129, 10084-+.
32. J. Dong, K. M. Solntsev and L. M. Tolbert, *J. Am. Chem. Soc.*, 2006, 128, 12038-12039.
33. A. Sinicropi, T. Andruniow, N. Ferre, R. Basosi and M. Olivucci, *J. Am. Chem. Soc.*, 2005, 127, 11534-11535.
34. M. E. Martin, F. Negri and M. Olivucci, *J. Am. Chem. Soc.*, 2004, 126, 5452-5464.
35. I. V. Polyakov, B. L. Grigorenko, E. M. Epifanovsky, A. I. Krylov and A. V. Nemukhin, *Journal of Chemical Theory and Computation*, 2010, 6, 2377-2387.
36. I. Polyakov, E. Epifanovsky, B. Grigorenko, A. I. Krylov and A. Nemukhin, *Journal of Chemical Theory and Computation*, 2009, 5, 1907-1914.
37. A. V. Bochenkova and L. H. Andersen, *Faraday Discuss.*, 2013, 163, 297-319.
38. C. R. S. Mooney, D. A. Horke, A. S. Chatterley, A. Simperler, H. H. Fielding and J. R. R. Verlet, *Chemical Science*, 2013, 4, 921-927.
39. D. Mandal, T. Tahara and S. R. Meech, *J. Phys. Chem. B*, 2004, 108, 1102-1108.
40. N. M. Webber, K. L. Litvinenko and S. R. Meech, *J. Phys. Chem. B*, 2001, 105, 8036-8039.
41. S. Olsen and S. C. Smith, *J. Am. Chem. Soc.*, 2008, 130, 8677-8689.
42. A. Toniolo, S. Olsen, L. Manohar and T. J. Martinez, *Faraday Discuss.*, 2004, 127, 149-163.
43. T. Mori and T. J. Martinez, *Journal of Chemical Theory and Computation*, 2013, 9, 1155-1163.
44. N. Y. A. Baffour-Awuah and M. Zimmer, *Chem. Phys.*, 2004, 303, 7-11.
45. W. Weber, V. Helms, J. A. McCammon and P. W. Langhoff, *Proceedings of the National Academy of Sciences of the United States of America*, 1999, 96, 6177-6182.
46. Y.-H. Hsu, Y.-A. Chen, H.-W. Tseng, Z. Zhang, J.-Y. Shen, W.-T. Chuang, T.-C. Lin, C.-S. Lee, W.-Y. Hung, B.-C. Hong, S.-H. Liu and P.-T. Chou, *J. Am. Chem. Soc.*, 2014, 136, 11805-11812.
47. M. S. Baranov, K. A. Lukyanov, A. O. Borissova, J. Shamir, D. Kosenkov, L. V. Slipchenko, L. M. Tolbert, I. V. Yampolsky and K. M. Solntsev, *J. Am. Chem. Soc.*, 2012, 134, 6025-6032.
48. M. Paolino, M. Gueye, E. Pieri, M. Manathunga, S. Fusi, A. Cappelli, L. Latterini, D. Pannacci, M. Filatov, J. Léonard and M. Olivucci, *J. Am. Chem. Soc.*, 2016, 138, 9807-9825.
49. S. Chatterjee and P. Karuso, *Tetrahedron Letters*, 2016, 57, 5197-5200.
50. S. Sharafy and K. A. Muszkat, *J. Am. Chem. Soc.*, 1971, 93, 4119-4125.
51. C. García-Iriepa, H. A. Ernst, Y. Liang, A.-N. Unterreiner, L. M. Frutos and D. Sampedro, *The Journal of Organic Chemistry*, 2016, 81, 6292-6302.
52. W. J. H. Ong, S. Alvarez, I. E. Leroux, R. S. Shahid, A. A. Samma, P. Peshkepija, A. L. Morgan, S. Mulcahy and M. Zimmer, *Molecular Biosystems*, 2011, 7, 984-992.
53. H. Hosoi, S. Hazama and Y. Takeda, *Chem. Phys. Lett.*, 2015, 618, 186-191.
54. R. Ando, H. Mizuno and A. Miyawaki, *Sci*, 2004, 306, 1370-1373.
55. A. Ericsson, J. Kowalewski, T. Liljefors and P. Stilbs, *J. Magn. Reson.*, 1980, 38, 9-22.
56. I. A. Heisler, M. Kondo and S. R. Meech, *Journal of Physical Chemistry B*, 2009, 113, 1623-1631.
57. D. Mandal, T. Tahara, N. M. Webber and S. R. Meech, *Chem. Phys. Lett.*, 2002, 358, 495-501.
58. B. Bagchi, G. R. Fleming and D. W. Oxtoby, *Journal of Chemical Physics*, 1983, 78, 7375-7385.
59. J. T. Hynes, *Annual Review of Physical Chemistry*, 1985, 36, 573-597.
60. M. J. van der Meer, H. Zhang and M. Glasbeek, *Journal of Chemical Physics*, 2000, 112, 2878-2887.

61. S. Takeuchi, S. Ruhman, T. Tsuneda, M. Chiba, T. Taketsugu and T. Tahara, *Sci*, 2008, 322, 1073-1077.
62. P. J. M. Johnson, A. Halpin, T. Morizumi, V. I. Prokhorenko, O. P. Ernst and R. J. D. Miller, *Nature Chemistry*, 2015, 7, 980-986.
63. J. Quenneville and T. J. Martinez, *J. Phys. Chem. A*, 2003, 107, 829-837.
64. L. Blancafort, *Chemphyschem*, 2014, 15, 3166-3181.
65. Q. S. Li, A. Migani and L. Blancafort, *Journal of Physical Chemistry Letters*, 2012, 3, 1056-1061.
66. A. Cembran, F. Bernardi, M. Olivucci and M. Garavelli, *J. Am. Chem. Soc.*, 2003, 125, 12509-12519.
67. I. Conti, M. Garavelli and G. Orlandi, *Journal of the American Chemical Society*, 2008, 130, 5216-5230.
68. S. Jenkins, L. Blancafort, S. R. Kirk and M. J. Bearpark, *PCCP*, 2014, 16, 7115-7126.
69. Q. S. Li and L. Blancafort, *Chem. Commun.*, 2013, 49, 5966-5968.
70. S. Olsen and R. H. McKenzie, *Chem. Phys. Lett.*, 2010, 492, 150-156.
71. H. E. Seward and C. R. Bagshaw, *Chem. Soc. Rev.*, 2009, 38, 2842-2851.
72. N. M. Webber and S. R. Meech, *Photochemical & Photobiological Sciences*, 2007, 9, 276-281

73. J. W. Park and Y. M. Rhee, *J. Am. Chem. Soc.*, 2016, 138, 13619-13629.
74. S. McConnell, R. H. McKenzie and S. Olsen, *The Journal of Chemical Physics*, 2015, 142, 084502.
75. L. X. Wu and K. Burgess, *J. Am. Chem. Soc.*, 2008, 130, 4089-4096.
76. P. Celani, M. A. Robb, M. Garavelli, F. Bernardi and M. Olivucci, *Chem. Phys. Lett.*, 1995, 243, 1-8.
77. G. W. T. M. J. Frisch, H. B. Schlegel, G. E. Scuseria, M. A. Robb, J. R. Cheeseman, J. A. Montgomery, Jr., T. Vreven, K. N. Kudin, J. C. Burant, J. M. Millam, S. S. Iyengar, J. Tomasi, V. Barone, B. Mennucci, M. Cossi, G. Scalmani, N. Rega, G. A. Petersson, H. Nakatsuji, M. Hada, M. Ehara, K. Toyota, R. Fukuda, J. Hasegawa, M. Ishida, T. Nakajima, Y. Honda, O. Kitao, H. Nakai, M. Klene, X. Li, J. E. Knox, H. P. Hratchian, J. B. Cross, C. Adamo, J. Jaramillo, R. Gomperts, R. E. Stratmann, O. Yazyev, A. J. Austin, R. Cammi, C. Pomelli, J. W. Ochterski, P. Y. Ayala, K. Morokuma, G. A. Voth, P. Salvador, J. J. Dannenberg, V. G. Zakrzewski, S. Dapprich, A. D. Daniels, M. C. Strain, O. Farkas, D. K. Malick, A. D. Rabuck, K. Raghavachari, J. B. Foresman, J. V. Ortiz, Q. Cui, A. G. Baboul, S. Clifford, J. Cioslowski, B. B. Stefanov, G. Liu, A. Liashenko, P. Piskorz, I. Komaromi, R. L. Martin, D. J. Fox, T. Keith, M. A. Al-Laham, C. Y. Peng, A. Nanayakkara, M. Challacombe, P. M. W. Gill, B. Johnson, W. Chen, M. W. Wong, C. Gonzalez, and J. A. Pople, *Gaussian 03, Revision A.1,Gaussian, Inc., Pittsburgh PA,*, 2003.
78. F. Aquilante, L. De Vico, N. Ferre, G. Ghigo, P. A. Malmqvist, P. Neogrady, T. B. Pedersen, M. Pitonak, M. Reiher, B. O. Roos, L. Serrano-Andres, M. Urban, V. Veryazov and R. Lindh, *Journal of Computational Chemistry*, 2010, 31, 224-247.

**Figure Legends**

**Figure 1. Electronic Spectra** (a) Absorption and emission spectra of **I** in a series of alcohol solvents (b) Effect of irradiation on the absorption spectrum of **I** in methanol showing the recovery from the photostationary state to dark equilibrium (PSS→Z) as a function of time and (inset) the kinetics of photoconversion Z→PSS (excitation at 365nm with irradiance of approx. 2 $mWcm^{-2}$). The solid curve is the fit discussed in SI 4. Data are presented in Table 1.

**Figure 2**. **Interconversion between ground-state isomers of I**. Relative energies are given in $kcal{\cdot}mol^{-1}$.

**Figure 3. The molecular HOMO and LUMO of *Z*-I**.

**Figure 4. Ultrafast Fluorescence**. Time resolved fluorescence decay profiles of **I** are shown for (a) a selected series of alcohol solvents measured at the peak of the emission (the data for HBDI in methanol are included to illustrate the remarkable quenching in **I**). (b) the plot shows the weak viscosity dependence (c) The wavelength dependence of the emission in propanol.

**Figure 5**. **Calculated excited-state decay pathway** for the *Z*-**I** and *E*-**I** isomers ($S_1$ and $S_0$ energies in eV) and representative structures along the path. (a) MS-CASPT2/ANO-S profile along the TD-CAMBL3LYP/6-311G** MEP from the Franck-Condon structure to the $S_1/S_0$ CI. The initial part of the path (0 - 5 a. u.) is shown with a different displacement scale for clarity. (b) Representative structures along the MEP showing the $C_5$-$C_{7'}$ and $C_{1'}$-$C_{7'}$ bond distances in Å and the values of the $\phi$ and $\tau$ angles. The variation in the angles $\phi$, $\tau$, $\rho$ and $\gamma$ (see Scheme 1 for definitions) along the decay pathway are shown in Figures S6 and S7.

**Figure 6.** (a) Superimposed structures of *Z*-FC (grey bonds) and *Z*-CI (green bonds). (b) Same for *E*-FC and *E*-CI.

**Scheme 1**

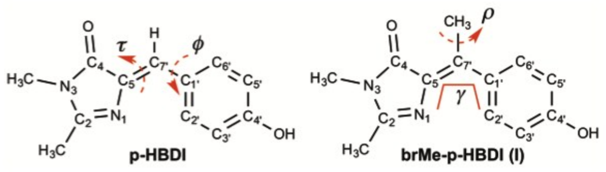


$\tau$ = $N_1$-$C_5$-$C_7$-$C_{1'}$ $\rho$ = H-$C_{Me}$-$C_7$-$C_5$
$\phi$ = $C_{2'}$-$C_{1'}$-$C_7$-$C_5$ $\gamma$ = $N_1$-$C_5$-$C_{1'}$-$C_{2'}$

**Figure 1**

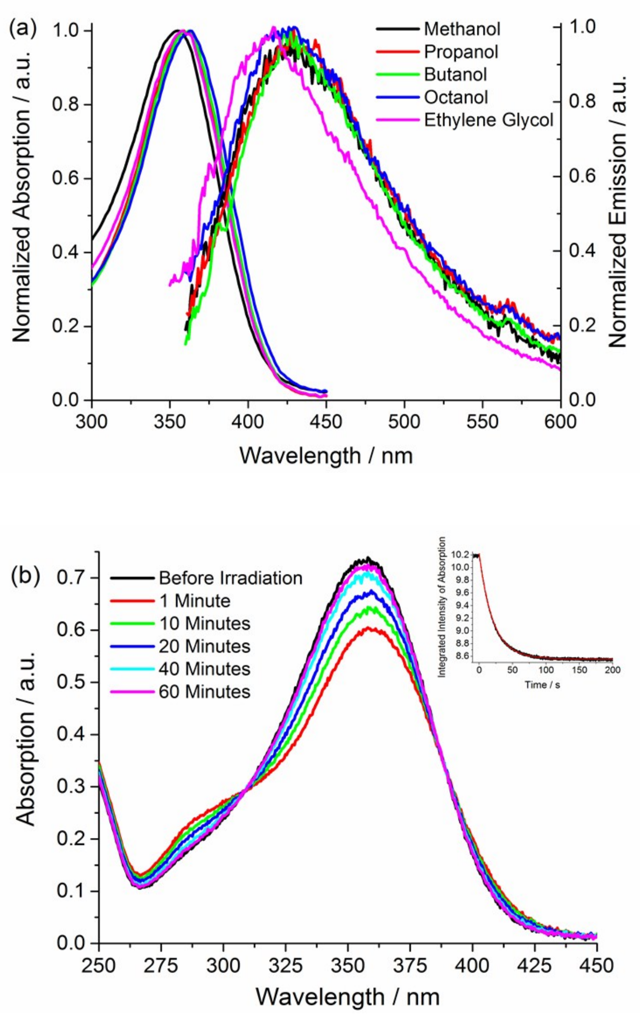

**Figure 2**

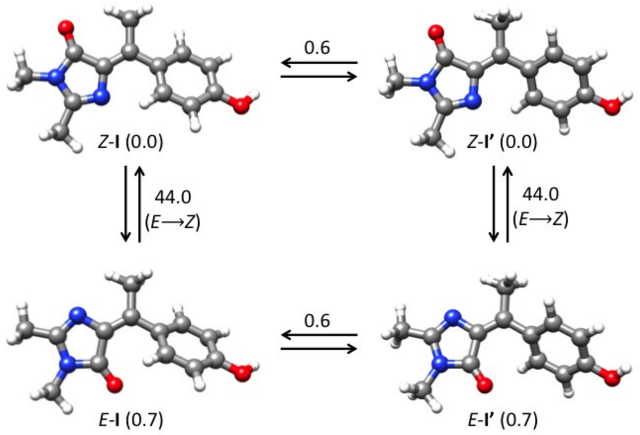


**Figure 3**

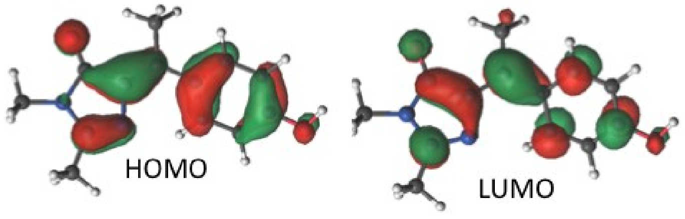

Figure 4

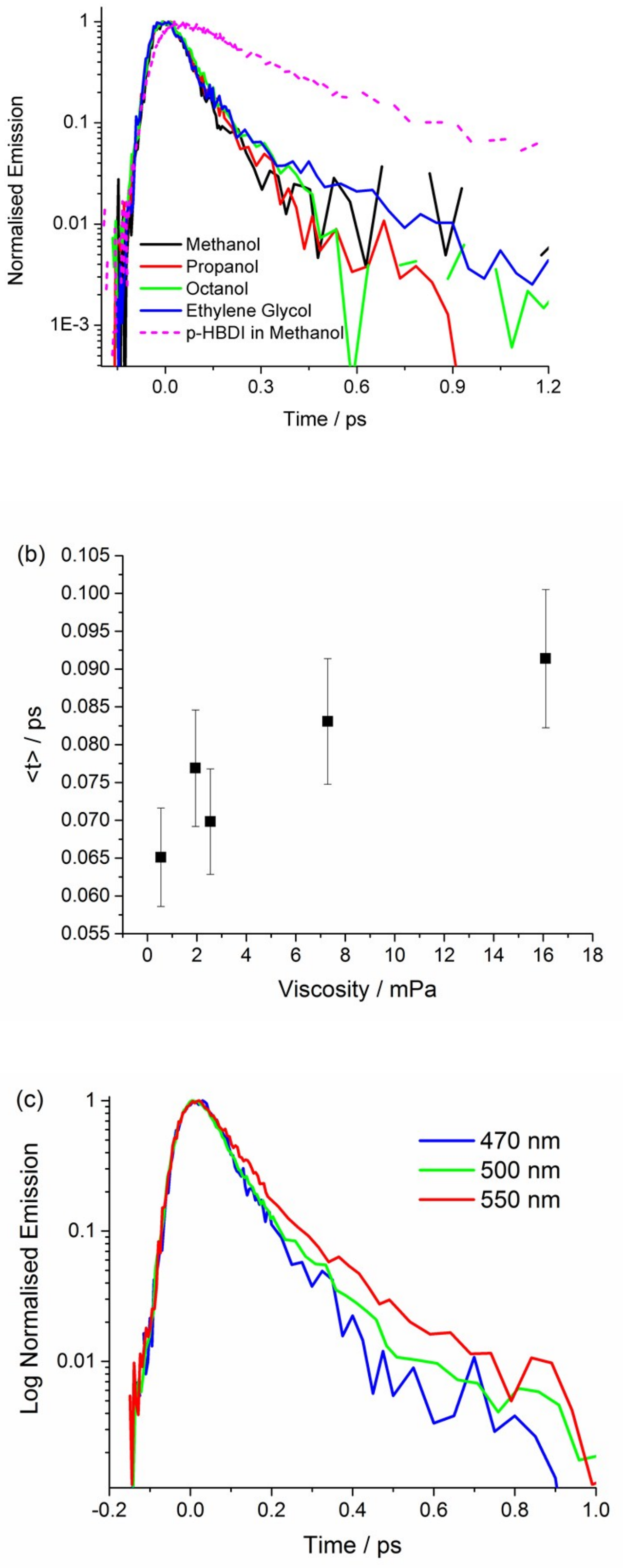
Normalised Emission
1
0.1
0.01
1E-3
0.0
0.3
0.6
0.9
1.2
Time / ps
Methanol
Propanol
Octanol
Ethylene Glycol
p-HBDI in Methanol
(b)
0.105
0.100
0.095
0.090
0.085
0.080
0.075
0.070
0.065
0.060
0.055
<t> / ps
0
2
4
6
8
10
12
14
16
18
Viscosity / mPa
(c)
Log Normalised Emission
1
0.1
0.01
-0.2
0.0
0.2
0.4
0.6
0.8
1.0
Time / ps
470 nm
500 nm
550 nm

**Figure 5**

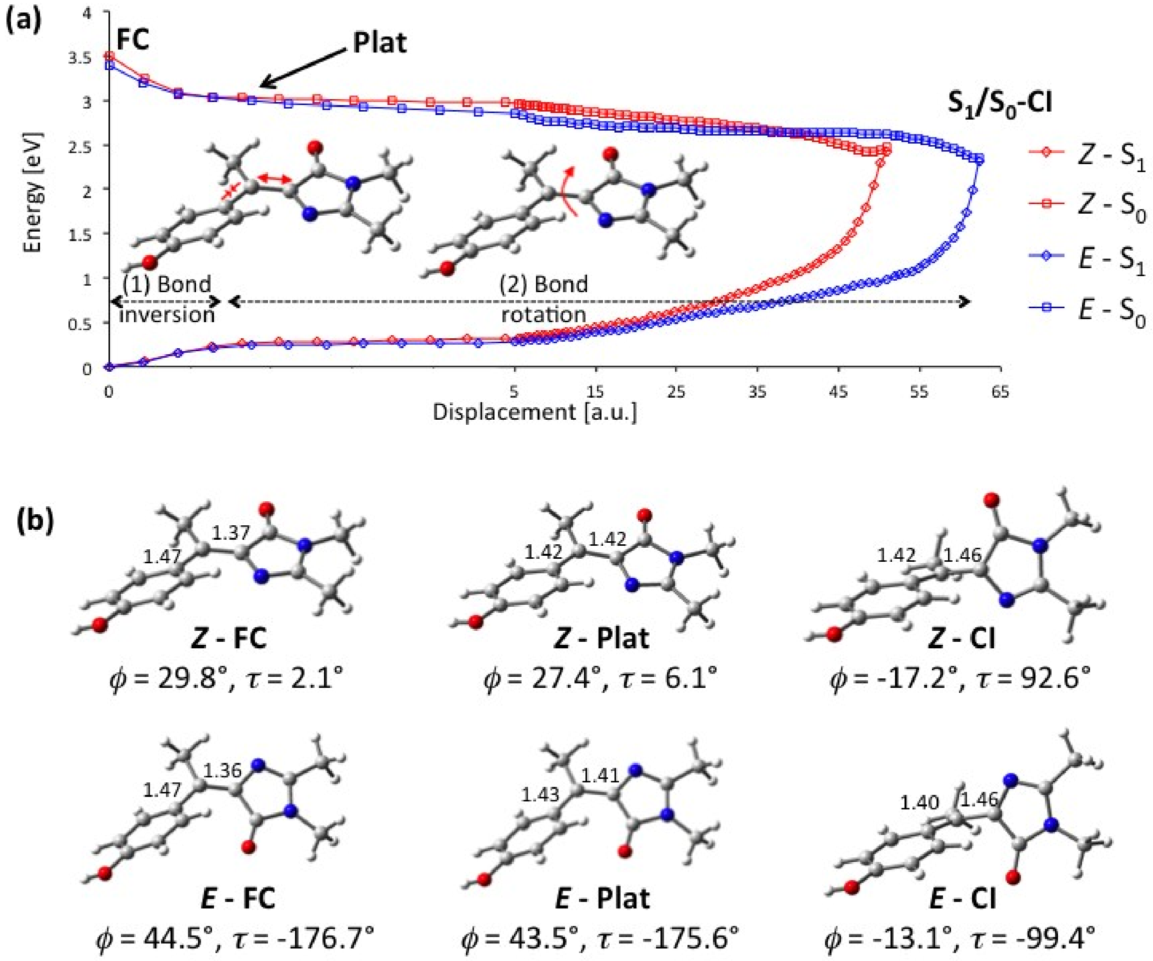


**Figure 6**

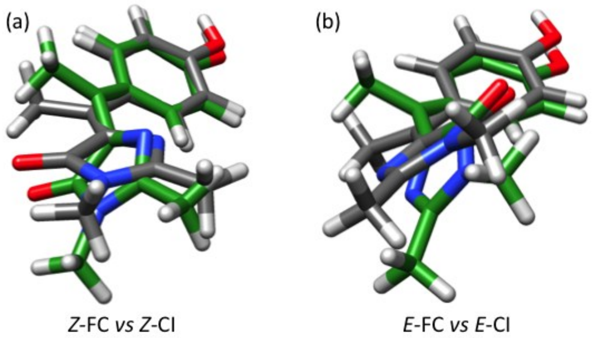

**ToC Figure**

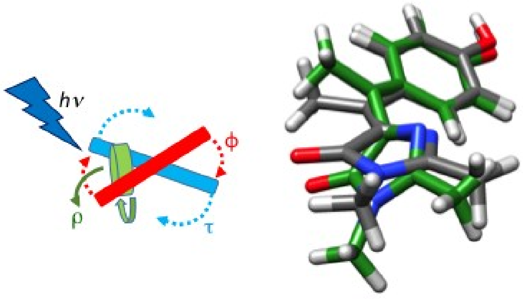


Dynamics of a nonplanar GFP chromophore are studied experimentally and theoretically. Coupled torsional motion is responsible for the ultrafast decay.
